# Strategies and perceptions towards flood control and waste management in Limbe city, Cameroon

**DOI:** 10.4102/jamba.v15i1.1390

**Published:** 2023-11-20

**Authors:** Mabel N. Wantim, Asong F. Zisuh, Ngankam S. Tendong, Roy L. Mbua, Emilien N. Findi, Samuel N. Ayonghe

**Affiliations:** 1 Department of Environmental Science, Faculty of Science, University of Buea, Buea, Cameroon; 2 Disaster Risk Management Unit, Faculty of Science, University of Buea, Buea, Cameroon

**Keywords:** floods, municipal solid waste management, perception, Benefit Value Tree, Limbe, Cameroon

## Abstract

Limbe city in Cameroon is prone to annual flood disasters. The Limbe City Council (LCC) has been making efforts to address the problem of floods and municipal solid waste management (MSWM). This article presents an: (1) assessment of the different strategies by the council towards MSWM and floods; (2) evaluation of public perception on waste disposal practices and (3) examination of the nexus between waste disposal and flooding. The methods employed included qualitative data collection through semi-structured interviews with the aid of questionnaires (open- and closed-ended questions) field observations and interview; and the use of the Benefit Value Tree method (BVT) to analyse sustainability strategies by LCC. The BVT method provided, the following scores for the four strategies used: sustainable basin management against floods and/or landslides = 7; mechanical dredging of river beds = 4; Keep Limbe Clean = 3; manual dredging and cleaning of rivers, gutters, culverts and bridges = 1. An evaluation of the municipal solid waste collection system revealed that (1) over 85% of plastic bins do not meet standard physical conditions; (2) bins at all collection points are insufficient for waste at such points and (3) residents engage in wanton disposal practices because of insufficient service coverage of residential areas. Field observations and interview with authorities established a clear connection between floods and poor waste management. In addition to existing efforts by LCC, it needs to upgrade and expand waste collection services through provision of numerous suitable bins and community awareness raising on the nexus between floods and waste disposal.

**Contribution:**

This is a transdisciplinary research which presents the constraints and challenges in waste generation and collection, its relationship with recurrent floods in Limbe city, and presents a way forward to improve on the situation using the BVT method.

## Introduction

Floods are devastating events and have been assessed to result in the highest number of casualties. Recent statistics show that 95% – 97% of fatalities from natural hazards are caused by floods in developing countries and characterised with highest economic losses of $250 billion worldwide over the last 15 years alone, when compared to all other disasters triggered by natural occurrences (Tariq, Farooq & Van De Giesen 2020:1). Across all continents, flood events have increased in frequency and severity. This increase is linked to a variety of causes including changes in weather and/or climate events, but equally from activities relating to urbanisation (Jha, Bloch & Lamond 2012). These flooding events have even taken major economies by surprise as was shown in the July 2021 flood event in Germany (Fekete & Sandholz 2021:1–20). Even though floods are a known natural hazard in Germany, the amount of precipitation and ensuing high death toll (over 180 deaths with over 40 000 affected people) and damages after the 14 July 2021 –15 July 2021 event came as a surprise (Fekete & Sandholz 2021:1–20).

In sub-Saharan Africa alone, 654 flood events have affected 38 million people, and resulted in about 13 000 deaths in the last three decades (Salami, Von Meding & Giggins 2017). Flood risks in African cities (e.g. Ibadan in Nigeria and Accra in Ghana) have been largely exacerbated because of anthropogenic influences such as rapid urbanisation, unregulated informal settlements characterised by low socioeconomic conditions and physically deficient structures on the low-lying floodplain areas, disregard of waste management, and poor maintenance of drainage systems (Salami et al. 2017; Yin et al. 2021). In recent decades, many factors such as rural-urban migration, rapid industrialisation, population explosion and lifestyle changes have led to a significant increase in the amount of municipal solid waste (MSW) production (Jaafarzadeh et al. 2020; Sakijege 2019:177–199).

Municipal solid waste is primarily composed of kitchen waste, yard waste, paper and cardboard, textiles, leather, rubber, plastic, glass, wood, metal and other materials (Zhang et al. 2021). This waste frequently leads to blockages in drainage systems and watercourses and causes practical negative effects on the environment such as urban flooding, pollution by discharge and leaching into surface water and groundwater, and the resulting risks to human health and property (Lamond, Bhattacharya & Bloch 2012). Low priority is often accorded to the management of solid waste in developing countries (Lamond et al. 2012) because of inadequate financial resources, low levels of enforcement of regulations and poor governance (Parrot, Sotamenou & Dia 2009:986–995; Tvedten & Candiracci 2018:631–646). According to the Resilient Cities Report (2017), poor solid waste management (SWM) can worsen the burden of urban flooding by blocking drainages and creating a fertile ground for disease vectors; hence considerations of water and SWM should be inseparable from urban adaptation strategies (Diaz, Eggerth & Golueke 2002). The 2012 World Bank report (World Bank 2012) estimated MSW generation levels to double by 2025.

Towns and cities in Cameroon exhibit the burdens of waste management which characterise so many African countries (Manga et al. 2008). Municipal solid waste management (MSWM) system in Cameroon faces significant challenges with respect to the different functional elements (Asong 2010; Parrot et al. 2009). In an attempt to handle this problem, the MSWM sector has experienced different policy actions such as the transfer of responsibility of MSWM within municipalities to councils. Following this transfer, several councils (e.g. Bamenda City Council found in the North West Region of Cameroon) have now taken full responsibility of MSWM within their municipalities, while others have contracted a third party. Furthermore, international agendas place MSWM within an interdisciplinary perspective. Such agendas include the Sendai Framework on disaster risk reduction (DRR) (Aitsi-Selmi & Murray 2015) and the Sustainable Development Goals (SDGs) (United Nations 2015) that emphasise on the need to ‘understand and manage risk’ rather than disaster and to build sustainable cities (SDG17). Also, the Leading Integrated Research (LIRA) for Agenda 2030 African 2017 projects stressed on the need to build resilient cities where a good understanding of the nexus between disasters, health and energy within cities is established.

Despite the awareness that some of the low-lying areas in Limbe city are not habitable, the population in the last two decades has adopted a resigned attitude towards this and has encroached on the steep hill slopes known for their catastrophic landslides (Ndille & Belle 2014:147) resulting in grievous consequences. Each year, between the months of June and August, often characterised by weeklong torrential rains, the city is submerged in water, above a metre deep in some localities, causing serious distress to the local population and great embarrassment to the municipal authorities and local government officials. According to statistics, each year about 100 homes are flooded and an average of 5 landslides occurs in the city (Lambi, Kometa & Fombe 2002; Ndille & Belle 2014). In some years, the situation became so critical that it required national emergency alerts – such as in 2001, 2007, 2013, 2014 and 2018 (Buh & Aka 2018; Cheo 2012; Ewoko 2010; Ndille & Belle 2014). The worst case scenario so far occurred in June 2001 where heavy rains led to a combination of floods and landslides and resulted in more than 30 landslide scars that destroyed 154 houses, killed 93 people and a significant number of livestock and rendered over 233 people homeless (Lambi et al. 2002; Ndille & Belle 2014). The 2007 and 2018 floods also recorded ~29 deaths and destruction of over 80 houses with major population displacement (Ndille & Belle 2014). Rise in flood risk in Limbe city is connected to climate change, uncontrolled dumping of waste in watercourses (Manaf, Samah & Zukki 2009; Zurbrügg 2003) and urbanisation which has led to the proportional increase of a catchment’s impermeable surface area (Roudier, Ducharne & Feyen 2014).

Poverty affects over 85% of the Limbe population (Awum, Bayie & Fonda 2001), as a result over 70% of the population of Limbe lives in make-shift temporal structures constructed on floodplains. These houses are found too close to the coastline or on hillslopes and under slum-like conditions, a situation of high vulnerability to floods (Ndille & Belle 2014). Municipal solid waste generated from these slums are mostly deposited in rivers and streams. Henry, Yongsheng and Jun (2006) cited cases in Kenya whereby Nairobi River and Nairobi Dam were polluted by MSW generated from the nearby slums. Previous studies on floods carried out in the Limbe Municipality were based on climate change, lack of embankments and a couple of anthropogenic factors and gave little or no consideration to the role played by MSWM practices and flood risk in the area. Understanding therefore the relationship between MSWM practices and flood risk is vital to evaluate the contributions of the practices to increased flood events.

Taking Limbe City Council (LCC) as a case study, it is important to evaluate existing projects within the aforementioned nexus and to develop strategies on how they can be improved upon. To accomplish such a task, it will also be important to investigate local perceptions about these projects. This study therefore sought to: (1) assess the different policy options and strategies used by the LCC towards MSWM and floods in the municipality; (2) investigate public perception on disposal practices within the municipality; and (3) evaluate the nexus between waste disposal and flooding in Limbe municipality.

## Research methods and design

### Description of study area

This study was carried out in Limbe municipality, located on the southern slopes of Mount Cameroon in the South West Region. Limbe city is situated between longitude 9º E and 13° E and latitude 4° N and 9° N. It is dominated by the equatorial climate, characterised by a mean annual rainfall of 2000 mm, with a mean annual temperature of 26.5 °C and an annual average relative humidity of 82.5%. It has a population of about 120 000 inhabitants with an estimated growth rate of 2.9% (BUCREP 2010). In 2007, the municipality was carved into three subdivisions namely Limbe I with head office in Bota, Limbe II with headquarters in Mokundange and Limbe III with headquarters in Bimbia, and with all having respective municipal councils. The city remains a major economic hub of the South West Region of Cameroon. Its location along the Atlantic coastline of West Africa does not only facilitate trading and transport between Cameroon and other countries especially Nigeria, but also makes it a fishing locality. It is host to Cameroon’s lone oil refinery SONARA and also hosts the headquarters of the Cameroon Development Cooperation (CDC), second biggest employer after the government. The City’s road network covers over 37 km of tarred roads with about 30 km of this constructed in the early 1980s. Because of its location and rapid urbanisation, there is a huge influx of people from rural areas and other neighbouring countries leading to an increase in population density. This has led to rapid urbanisation with people building on hill slopes, risk zones, and river banks.

**FIGURE 1:**
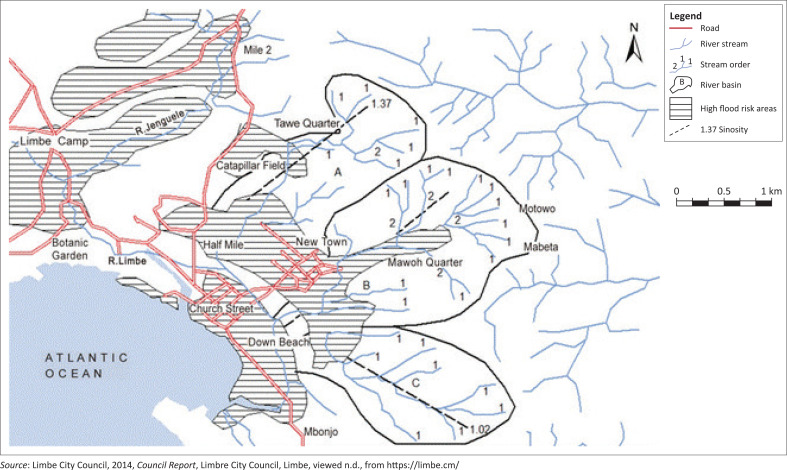
Map of Limbe situating the residential areas used in this study.

As reported by Che et al. (2012), the geology of Limbe is mainly igneous and consisting of different varieties of rock type including porphyritic basalts, pyroclastic, lahar, alluvial deposits, beach sand-shingle, and pillow lava. Soils derived from porphyritic basalts in Limbe are mottled, reddish brown, yellowish brown and/or pale yellow clayey silt, silt and clays with diverse physical and chemical properties. The city is drained by a number of permanent and intermittent streams, but the main ones that flood the city are the Limbe River, Womange River and Njenguele River, which are all situated in Limbe I sub-division. For this study, data were collected from the following residential areas within the municipality: Mowoh, Motowoh, Clerk’s Quarters, Newtown, Church Street, Lower Towe, Gardens, Mile 2, Middle Farms and Bota (Figure 1). However, for areas like Lower Towe, Gardens, Middle Farms and Bota that rarely experienced flooding, investigations were done only on aspects of MSWM.

### Study design

**FIGURE 2:**
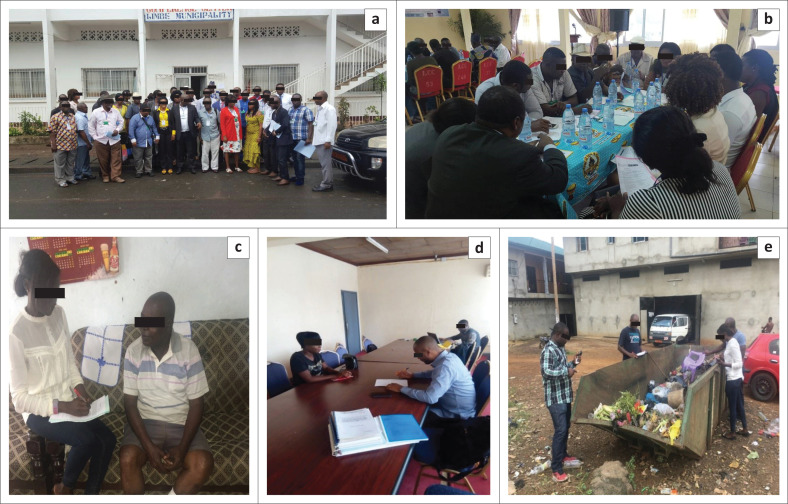
Photographs taken from 2017 to 2019 in Limbe showing (a) participants at the workshop on floods; (b) focus group discussion session at the workshop; (c) questionnaire administration in the field for the selected residential areas; (d) key informant interview with Limbe City Council personnel and (e) data collection in the field by direct observation and estimation made by research team members.

The objectives of this article were realised by utilising both transdisciplinary and descriptive research approaches. In this approach, three broad lines of activities were undertaken: (1) workshops with focus group discussion sessions, (2) fieldwork and (3) data analysis. An initial workshop with focus group discussion sessions was held between the research team (made up of earth, environmental and social scientists) and some participants comprising persons from administration, councils and community groups (non-scientific) (Figure 2a and Figure 2b). The aim of this workshop was to enable the research team to interact with local actors so as to get their perceptions of the waste management and flooding problems within the city of Limbe in a bid to better refine the objectives of the study. An interview guide with defined questions was used for this session to facilitate data collection. During this session, some key problem areas were brought up and participants gave their views on each of the key areas.

### Data collection

Limbe city hosts a population of ~120 000 inhabitants (BUCREP 2010). Sample size was calculated using the Slovin formula with an assumed precision of 5%:


$$n=\frac{N}{1+N\left(e^{2}\right)}$$ [Eqn 1]

where *n* = maximum sample size:

*N* = population of Limbe city

*e* = precision (5%; conventional)

This gave an approximate sample size of 400 participants. However, in this study, only 226 questionnaires were administered. This is because the study was limited only to residential areas affected by flood events in the city which summed up to six. Furthermore, within the affected localities, not all the households participated in the study. Only those who willingly compiled were interviewed. This study is part of the project activities carried out during the LIRA 2030 research activities; thus the ethical clearance used in the study by Wantim et al. (2022:428) was also applicable for this study.

Fieldwork involved the following activities: administration of questionnaires to households, making observations at waste collection points within the city, and interview of some local council staff (Figure 2c and Figure 2d). Two sets of questionnaires were developed, and both semi-structured and structured questions were used. The first set of questionnaires was concerned with waste management issues in the municipality and the second was related to floods. The questionnaires were administered to some households in the selected residential areas as mentioned. The process began with a reconnaissance survey that comprises of fieldwork to identify houses that were regularly and directly affected by flooding in six (Mowoh, Motowoh, Clerk’s Quarters, Newtown, Church Street and Mile 2) of the selected neighbourhoods (Figure 1). More so, the willingness of such households was sought through the informed consent that was included as part of the questionnaire as per the University of Buea guidelines and also explained orally to non-literate participants to participate in a questionnaire survey. Given that for the six residential areas considered, the total number of houses directly affected by floods per area was not more than 150 (mostly along stream banks), and based on the willingness to participate, we set a minimum of 40 questionnaires per neighbourhood. Households in flood-prone areas participated in both surveys, while those in non-flood-prone areas (four localities in total: Lower Towe, Gardens, Middle Farms and Bota) did not participate in the questionnaire on floods. A total of 226 questionnaires were administered for both sets over a period of 2 weeks.

An assessment of public perception and awareness about some aspects of the MSWM system in Limbe included the frequency of collection, storage and disposal practices, awareness and knowledge on MSWM. This is important because the success of any MWSM system depends a lot on public participation. Thus in order to make an appraisal of the physical conditions at secondary waste collection points, we undertook a patrol of the city, stopping at almost all main secondary collection points within the selected areas for this study. At each stop, a visual inspection was made to establish the following: (1) the presence and/or absence of a collection container; (2) if present the physical conditions of the container(s) and (3) the types of waste dropped at collection points (Figure 2d). Some key personnel of LCC such as the mayor were interviewed with the aid of an interview guide on issues relating to waste and flood management for the city.

In the last phase during questionnaire administration, respondents were further requested based on their experience to past flood events to give their perception of the severity of major floods from 2001 to 2018 since the study took place between 2017 and 2019. An understanding of the dynamics and impacts of flooding in Limbe municipality based on local community perception is important in developing strategies for better inclusive flood management. Thus, respondents were asked, based on their perceptions, to rank the major floods, i.e. 2001, 2004, 2007, 2014 and 2018, in order of their severity on a scale of 1–3 corresponding to very severe, severe and not severe, respectively. A qualitative criterion was used here where very severe (1) corresponded to floods that caused significant environmental and infrastructural disruption, including death, while not severe (3) referred to floods that had no significant consequences. The data obtained were treated using simple statistics in MS Excel to get total responses for the given years in the studied neighbourhoods.

### Data analysis

Data obtained from this study were analysed using the Statistical Package for the Social Sciences (SPSS) 16.0 (SPSS Incorporated 2007) and graphs were produced with the aid of MS Excel. Deductive inferences were made from field observations and interviews. The Benefit Value Tree (BVT) method of Costa, Da Silva and Correia (2004) and adapted by Andersson-Sköld and Nyberg (2016) was used in evaluating the effectiveness of different flood control and hygiene measures utilised by the council. In the assessment, the costs of the flood control measures were compared to the risk reduction effectiveness as well as other impacts of the risk reduction measures under investigation. The BVT consisted of three main non-economic components (key benefit dimensions), environmental impacts, social impacts and technical effectiveness, that were analysed in relation to the costs of the flood control measure being assessed. In this study, we made use of a semi-quantitative scale. The scale ranged from -2 (very negative impact on the ‘benefit’ component, that is, significantly more negative than -1) to +2 (very positive impact on the component, that is, significantly more positive than +1), where 0 implies no or no significant impact. A total of the values for each measure was recorded, and these values were used to rank the risk reduction measure.

### Ethical considerations

Ethical clearance to conduct this study was obtained from the University of Buea Department of Environmental Science, Faculty of Science.

## Results

### Perception and awareness of municipal solid waste management practices by households

**FIGURE 3:**
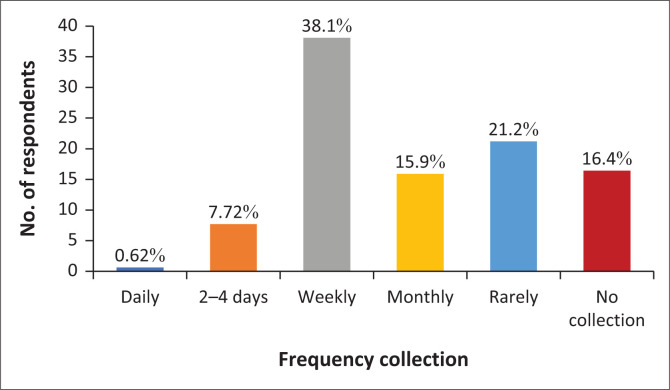
Frequency of collection of household waste as per the respondents in Limbe.

Findings on the responses of households on frequency of waste collection in Limbe within the past 3 years (2015 to 2018) ranged from collection within a few days to monthly and in worst case scenario rarely (Figure 3). The established frequency of door-to-door waste collection for the municipality was at least once a week.

The results revealed that waste collection for the selected residential areas has been poor considering that the response options comprising monthly, rarely and no collection constituted 50% of the responses (Figure 3). From field interviews conducted, it was realised that since 2017, the frequency of collection has been on a decline with this being blamed on the insufficient number of workers and vehicles available for waste collection. Regular collection with frequencies of up to twice a week occurred mostly in the central parts of the municipality characterised with mainly paved routes. Frequency of collection in more remote and peripheral areas is less and in some cases there is no collection at all. This leaves residents in such areas with the sole option of wanton disposal of waste. Field observation showed that waste from such peripheral areas constitutes a major load for streams running through such areas, and hence a contributor to blockages of channels thus triggering overflows.

### Waste storage and disposal practices within the municipality

**FIGURE 4:**
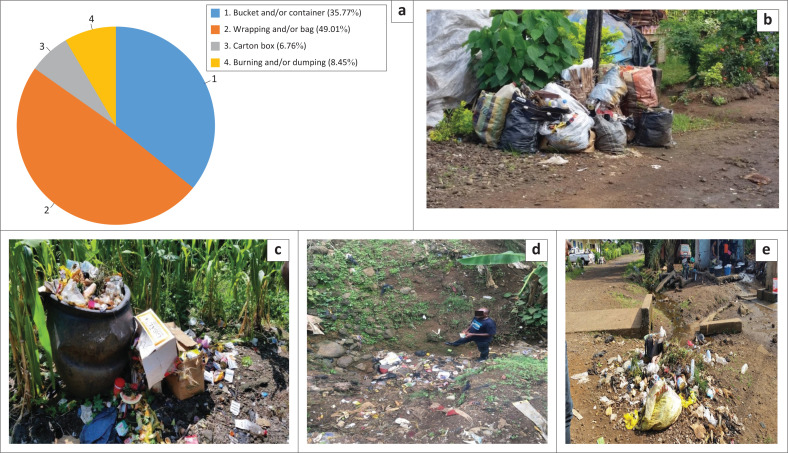
Household waste storage/disposal options in Limbe as per (a) respondents; (b) field observation; (c) waste dump with cartons used as household dumps; (d) an intermittent stream channel (about 4 m deep and 2.5 m wide) along whose channel municipal solid waste is regularly disposed in and (e) waste disposal besides a bridge over a stream channel.

Based on the respondents observations, it was realised that household waste storage containers ranged from plastic wrappings, nylon bags, cartons and buckets (Figure 4). Field results revealed the following with respect to storage devices: polythene bags 49.0%, buckets 35.8%, carton Box 6.8% and burning and/or dumping 8.45% (Figure 4a). Wastes in these devices can be stored at households for up to a week. In cases where households are located along collection routes, they are placed besides the road for onward collection (Figure 4b).

From field observation, upon collection, the contents of the storage devices are manually emptied into the collection trucks – a very labour-intensive exercise. Although residents make use of the aforementioned storage devices, they are, however, not sustainable options. On rainy days, cartons lose their strength and thus cannot be conveniently lifted (Figure 4c). In some cases, the device is dropped alongside the wastes into the truck. Thus, based on cost, health and convenience dimension, the storage practices are not sustainable. Where the neighbourhood is not served with collection option, residents choose to either dump their wastes in nearby streams or burn them (8.5% respondents). Field observation shows that disposal of wastes into streams (Figure 4d) is not only a function of not being served with collection option, it was also observed that houses besides streams are a little far from collection routes (in some cases over 100 m), for example, we observed along a street which stretched about 100 m, five such piles less than 8 m from residential houses. In this particular case, just a single pile was covered with a mosquito net and the residents said they did so in a bid to keep it free of vectors and pests. More so, such piles are also not uncommon besides streams (Figure 4e).

### Awareness and information on proper waste management

In this study, it was observed that 40% of respondents have some formal information channel on proper waste management that ranged from media (22%), seminar and/or workshops (3%), social meetings (8%), social media (2%) and institutions (5%). The percentage (22%) of those who tend to have formal information get this information through media outlets such as TV and radio programmes. Other sources of information are institutions including social meetings, hospitals and schools. On the other hand, about 60% of respondents are not linked to any formal information sources on waste management.

### Waste collection in Limbe Municipality

**FIGURE 5:**
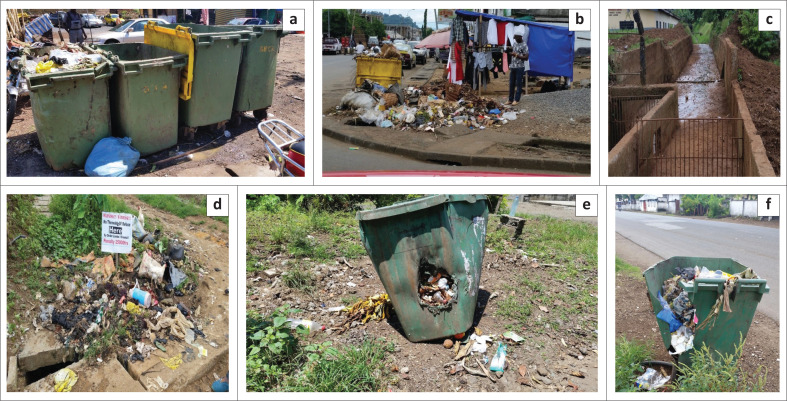
Field photographs in Limbe city showing (a) municipal solid waste (MSW) collection point with plastic bins where just two have wheels; (b) metal collection bin (yellow colour); (c) rectangular cemented shallow gutter where people use to illegally dump waste in an area characterised by regular floods that had recently been cleaned manually; (d) unauthorised dump site as indicated by the sign post placed by the council with even a fine for defaulters located over a drainage system; (e) MSW collection bin with no wheels, no lid and a hole on its side as a result of fire and (f) MSW collection bin in a broken state.

In Limbe, based on field observation, primary waste collection is done by the public wherein they bring waste from their individual residents to secondary waste collection points. At secondary collection points, different types of bins have been placed (Figure 5a). There are typically three types of secondary collection bins that are used in Limbe: (1) plastic bins of different sizes; (2) metal containers and (3) rectangular cemented shallow gutters (an area of about 7.5 m^2^ and shallow depth of less than 50 cm) (Figure 5a, Figure 5b and Figure 5c). The bins are mainly 120 L, 360 L and 770 L in volume. while the metal container is 9 m^3^ in volume. Another collection facility, which is not a device per se, are unauthorised spots where residents drop their wastes on the ground surface for onward collection and transportation by waste management agents of the municipality (Figure 5d). In waste management, a major consideration is to reduce to a very high extent the interaction between the waste and the environment.

Collections bins are generally made to be pushed on roller wheels to collection trucks for emptying of contents. Thus, the presence of functional roller wheels on bins and good physical condition of bins are important variables in reducing collection time. In the observed cases, 28% had wheels while the rest either had no wheels or partially broken wheels (Figure 5a). In terms of the physical condition of bins, less than 10% are in good shape. They are either broken or have holes as a result of fire incident caused by street cigarette smokers or by mere fact that people drop kitchen waste containing hot charcoal in them (Figure 5e and Figure 5f). In some instances, the bins are empty or with wastes found lying on the ground (Figure 5b). This implies that despite the fact that councils are operating under tight budgets, time losses in operations as a consequence of poor physical state of bins further constrain the waste management system of the municipality.

### Strategies towards effective waste management and floods control

The strategies put in place by the LCC for flood control and waste management can be sub-divided into short-term measures, medium-term measures and long-term measures (Table 1).

**TABLE 1:** Flood control measures put in place by the Limbe City Council summarised from council reports and interviews.

Measures	Description
Short term	**(1) The City Council engages seasonal workers**
	They manually dredge part of the rivers which are humanly accessible and other drainage structures (gutters, culverts and bridges). Clean vegetation found in the river beds and river banks to facilitate the passage of runoffs into the streams and rivers.
	**(2) Bimonthly ‘Keep Limbe Clean’**
	Ensure that households clean their surroundings including gutters and drainages to permit the free flow of runoffs.
Midterm	Mechanical dredging of the river bed in areas which cannot be done manually assisted by Minister of Housing and Urban Development (MINDHU). Census on the directives of Minister of Housing and Urban Development to identify all those who have built on hill slopes, river banks and other risk zones. Cutting some areas of tarred roads to widen the culverts and thereby increase the volume of water to be canalised (e.g. the case of community field roundabout, behind old police barracks and in mile I between CDE and comprehensive high school).
Long term	**Sustainable Basin Management Project against floods and/or landslides**
	Creating channels to the sea through the streams and rivers of the municipality. Committing professional hydraulic studies for permanent solutions that will curb floods and other disasters in the municipality.

#### Short-term measures

**FIGURE 6:**
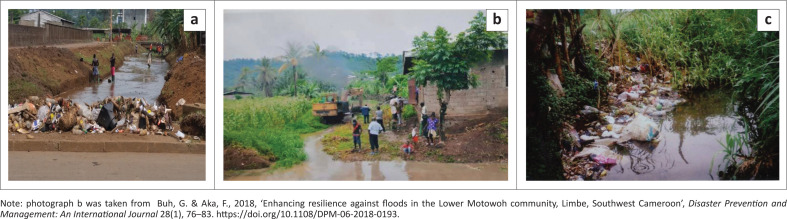
Photographs showing (a) widening of a stream channel within Limbe city in 2019 during period of fieldwork; (b) Dredging of Njenguele River in 2016 and (c) The state of Njenguele River as observed during fieldwork in 2019.

For the implementation of the short-term measures (Table 2), the City Council engages seasonal workers who manually dredge part of streams which are humanly possible and other drainage structures such as gutters, culverts and bridges (Figures 5c and 6a). These seasonal workers also clean vegetation found in the river beds and banks to ease flow as well as facilitate the passage of runoffs into the streams. The average annual cost for this exercise since 2012 stood at about 108 000 US dollars with projections increasing since then. The BVT assessment (Table 2) indicates that the measure contributes to improve channel flow, thereby reducing the chances for floods. There is also great awareness and acceptance of this measure among the inhabitants of the city. Given that the execution of this measure is mainly labour-intensive and manual, it poses health risk to those directly involved in the exercise. Considering environmental and social values as well as the technical complexity of the intervention, the result based on the BVT assessment is slightly positive (+1).

**TABLE 2:** Summary of results from applying the Benefit Value Tree method on the flood control measures.

Measure	Flood control measures	Benefits	Benefit value	Cost	Cost value	Sum
Short term	Manual dredging and cleaning of rivers, gutters, culverts and bridges	Enhances free flow of water in streams. Directly reduces the chances of floods and their severity. General public acceptance and participation. Provides temporal job opportunities. Little or no technical complexity.	6	Poor disposal of the dredged materials along the banks hence a temporal solution. Labour-intensive, time-consuming and financially costly. Possibility of health risks. Intervention is annual hence the problem of sustainability.	−5	1
	Keep Limbe Clean	Addresses the waste management problem at the source. Eases circulation of runoffs, streams. Wider public acceptance and participation. It has aesthetic benefits. Little or no technical complexity.	7	Little or no negative environmental impacts. Slows down economic activities of the day. Part of population not in support because of impact on economic activities. Intervention is temporal hence the problem of sustainability.	−4	3
Medium term	Mechanical dredging of river beds	Gains in agricultural land less likely impacted by flood events. Intervention contributed to reducing the flood risk but only in the short run. Reduced accumulation of contaminants in groundwater because of fewer flood events and shorter duration. Wider public acceptance and participation. Provide minor jobs in the short term. Excavation and/or dredging, relatively low technical complexity and frequency.	10	Intervention may result in sedimentation elsewhere along the river system. Intervention per se has an impact on the natural environment (its geology and habitat) because of the reduced riverine area. Logistics concerns and emissions (GHG) because of transport of dredged materials. The intervention costs 151 757 550 for every 2 years which is fairly too high for the municipality despite low complexity. The intervention requires machineries and transportation of the masses, which will use energy given that the measure is medium term.	−6	4
Long term	Sustainable basin management against floods/landslides	Will lead to permanent solutions that will curb floods and other disasters in the municipality. The identification, demolishing of settlements and/or relocation of people from risky zones curb the production of household waste and other debris that create downstream blockages. It uses the most environmentally friendly methods. It prevents future loss of lives and properties from the disaster. Given its sustainable nature, it easily attracts external funding and support. It leads to efficient use of resources and also leads to the protection of natural resources. It makes use of scientific and expertise methods before implementation making it highly efficient. It generally does not require maintenance as it is sustainable.	10	It has little or no negative environmental impacts. There is lack of awareness. A measure like this can be very costly. It is so complex and takes time.	−3	7

Another short-term measure put in place with the assistance of the civil administration is a bimonthly ‘Keep Limbe Clean’ exercise involving the entire population. During this exercise which takes place every first and third Wednesdays of every month, the services of hygiene and sanitation of the city and sub-divisional councils go round to ensure that households clean their surroundings including gutters and drainages to permit the free flow of runoffs. Applying the BVT to this intervention gives a positive outcome (Table 2).

As shown in Table 2, all the environmental aspects were positive (3), and if there had not been a conflict of interest between officials and the public regarding the temporal shutdown of business places during this exercise, the result would have been even more positive.

#### Medium-term measures

For its medium-term strategy, the LCC has identified certain areas in the city whereby structures such as culverts constructed in the early 1980s have become small and insufficient to canalise runoffs which empty themselves into the main streams. In such areas more technical work (mechanical dredging) is being done to increase the sizes of these structures (Table 1). Typical areas for this mid-term strategy include Limbe Community Field roundabout, behind former Police Barracks and in Mile I between Camerounaise des Eaux office and Comprehensive High School. Contracts for these projects stand at 163 330 US dollars.

Applying the BVT method to evaluate mechanical dredging of streambeds gave a positive outcome (Table 2). This intervention results in positive impacts in agriculture and it reduces floods and hence a reduction of land to be covered with flood water. It also contributes to improve stream water quality as streams would not tend to experience any stagnation of flow. The public strongly agree with this measure as well as participate financially in the realisation. A typical example is the dredging of the River Njenguele in 2016 whereby the community contributed 35% of the funds to hire the machineries needed for the exercise (Figure 6).

During fieldwork, some residential neighbourhoods like Lower Motowoh attested that these interventions in flood control are really beneficial (though emphasising on the short-term nature) as no major floods were experienced in their neighbourhood in 2016 and 2017 after the dredging of the Njenguele River. The measure is also regarded as not being complex. The intervention is, however, also expected to result in unwanted, possibly severe, impacts on the geology, and the intervention will have negative impacts on the ecosystem and habitat because of the reduced river area and the dredging (Table 2). In total, the result of this intervention is positive (+2) if all environmental and social values are summarised and even higher (+5) taking into account the level of protection and the low technical complexity of the intervention.

Another medium-term measure is the mechanical dredging of the riverbed in areas which cannot be done manually. This is usually done in the dry season before the rains at least once in 2 years. The LCC has submitted studies for assistance to the Ministry of Housing and Urban Development (MINDHU) which stands at 151 757 550 FCFA ($241837.80) for the dredging of Njenguele and the Limbe Rivers (Table 2). As another medium-term measure which is ongoing, the LCC carries out census on the directives of His Excellency, the Minister of Housing and Urban Development to identify all those who have built on hill slopes, river banks and other risk zones (Table 1). The purpose is to arrive at the cost of demolition and/or relocation of these people, some of whom produce household waste, debris and other vegetation which create downstream blockages.

#### Long-term measures

Regarding the long-term measures, Limbe has a peculiar characteristic because of its topography. It is nestled between Mount Cameroon with other surrounding hills and the Atlantic Ocean, and this gives Limbe a basin-like morphology (Figure 1). From this characteristic coupled with the heavy rains, water from the hills is to be channelled to the sea through the streams and rivers of the municipality. But in most cases, when it rains heavily, the sea level increases with huge sea waves pressurising the already overstretched rivers which are supposed to empty themselves into the sea, thereby causing floods which at times are accompanied by disasters.

In this regard, the LCC solicits a sustainable basin management against floods and/or landslides that includes creating channels to the sea through the streams and rivers of the municipality and committing professional hydraulic studies for permanent solutions that will curb floods and other disasters in the municipality (Table 1).

Applying the BVT in this way on the sustainable basin management measure resulted in a very positive outcome (7) (Table 2). All the environmental aspects were positive (4), and if it was not for the lack of awareness of this measure by the public and its monetary cost, the result would have been even more positive.

The grading presented in Table 2 was determined based on the qualitative impacts and their relative magnitudes described in the survey and interviews, and some documents provided by the council. The reason for the semi-quantitative and the discussion-based approach is the lack of relevant information, especially in-depth and quantitative information, because of lack of structured processes and documentation already in the decision and follow-up processes, which has also been found as a general obstacle in Cameroon risk management.

This grading as shown in Table 2 ranked the sustainable basin management against floods and landslides as the best option (total value of 7) to permanently curb floods in the Limbe municipality and manual dredging of rivers (total value of 1) as the least considering the environmental, socioeconomic and technical aspects.

### The connection between floods and waste management in Limbe

**FIGURE 7:**
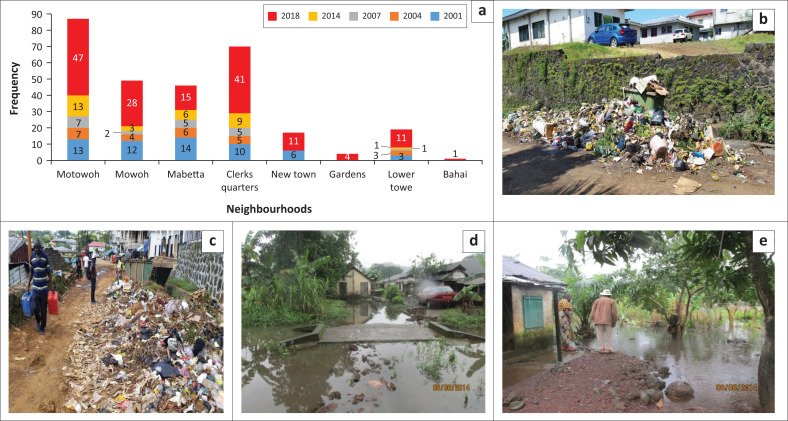
Flood and waste management in Limbe showing (a) the degree of perceived severity of the major floods in the last two decades in different neighbourhoods; (b) surface dump in residential areas with small-sized bins and delayed collection; (c) waste collection point at Motowoh with a small-sized container in relation to the population and hence heavy littering. Notice that the collection container is lying within a drainage path; (d and e) flooding at Clerk’s Quarters in 2014 caused by poor drainage systems and indiscriminate waste disposal that has clogged the available drainage systems.

Based on key informant interviews and discussions with the technical service of the LCC as well as from field observations, within the past two decades, the 2001 and 2018 floods had the greatest effects on eight affected neighbourhoods in Limbe (Figure 7a). Effects of floods in Limbe have mainly been disruption of services, physical damages to building infrastructure and death of humans (generally less than a few tens). Of the eight neighbourhoods mentioned, Motowoh, Mowoh, Mabetta and Clerk’s Quarters (Figure 1) are typical flood-prone areas of Limbe. From the participants’ responses, it was obvious that there is full agreement that the floods of 2001 and 2018 had the highest severity. The responses demonstrate a startling trend in that there is some unanimity on severity for the respective years. In 2001, for instance, Limbe lacked a good waste collection body. Municipal efforts towards this regard were not effective enough. The less severity and/or extent of the 2007 flood within mainland Limbe could be a further evidence of the change in the trend with the coming of the waste collection company HYSACAM. On the other hand, poor waste management aggravated the severity and/or extent of the 2018 floods in that through increased population and urban sprawl, the effectiveness of the waste collection process has been reduced (Figure 7b).

Another observation is that those living in these flood-prone areas tend to have a better memory of past floods than those living in non-flood-prone areas. Recently floods have been most severe at Motowoh and Clerk’s Quarters. In Motowoh, the main problem results from river channel blockage caused by indiscriminate dumping of refuse into the Njenguele River (Figure 6b). Equally, the waste collection done at Motowoh is not enough for its large population meanwhile waste bins are poorly used with wastes ending on the ground from where they are easily washed by runoff into the drainage systems thereby contributing to flooding (Figure 7b and Figure 7c). At Clerk’s Quarters, there are insufficient drains to handle the floodwaters (Figure 7d and Figure 7e). Poor waste management thus just aggravates the situation when the waste ends up in the already narrow drains (Figure 7d). This can explain the reason why flooding is comparatively more severe in Clerk’s Quarters. Mabetta (Figure 1 and Figure 7a), for instance, has the lowest frequency of waste collection (rarely), yet it has just a medium rate of severity while even though Motowoh has one of the highest rates of waste collection (weekly), it registers the highest occurrence of floods. This therefore shows that the rate of flooding is not dependent on the frequency of waste collection. This implies that waste management’s contribution to flooding is not solely dependent on the frequency of waste collection but probably through other reasons like poor disposal at the level of the household and poor drainage systems.

These observations on the severity of floods for some selected years tie with records obtained from municipal authorities. Municipal authorities attribute severity of floods in some areas to poor waste management. Thus, wanton disposal of waste in the presence of heavy precipitation will trigger floods. In 2001, for instance, Limbe lacked an efficient waste collection system. Clogged channels with waste were very common and the floods for this year were also severe. By 2007, the severity and/or extent of the floods had reduced. This could be attributed to the fact that the council had contracted a waste management company (HYSACAM) charged with the collection and disposal of waste for the city. By 2018, rapid increase in population and urban sprawl had started constraining the effectiveness of the waste collection system. More so, the state of collection vehicles has not been the best in terms of age and functionality.

From Figure 6a, it is seen that there are fewer responses for the following residential areas: New Town, Gardens, Lower Towe and Bahai. Two factors influenced this outcome, namely, the relief of these areas and the tempo spatial scale of their development. New Town and Gardens (Figure 1) are characterised by relatively higher relief than the other areas. However, Lower Towe, though on a relatively high to gentle relief, is characterised by more recent urban development with fewer residents. In some of the flooded areas, residents along the banks of streams suffer the impacts more. Thus, for areas like Gardens and Bahai with no streams, flooding is most often limited to standing water, which may not have significant environmental impacts because of flow velocity, but rather recesses or dries off after a while.

## Discussion

The results revealed that waste collection for the selected residential areas has been poor and on the decline since 2017. Household waste storage containers ranged from plastic wrappings, nylon bags, cartons and buckets, and the type of storage device is strongly dependent on individual households and not sustainable in most instances. Where the neighbourhood is not served with collection option or in cases where people reside beside streams and rivers, they choose to either dump their wastes in nearby streams or burn them. The study further revealed that 40% of respondents have some formal information channel on proper waste management, whereas the vast majority (60%) are not linked to any formal information sources on waste management, thereby promoting unstainable waste disposal practices. In Limbe city, primary waste collection is done by the public while secondary waste collection is done by the LCC. Long-term strategies such as the sustainable basin management against floods and landslides proved to be the best option to permanently curb floods in the Limbe municipality, while short-term measures such as manual dredging of rivers were graded as the least considering the environmental, socioeconomic and technical aspects involved.

From 2010 till date, the frequency of waste collection has experienced some changes. The result of this study is contrary to previous studies by Asong (2010), which indicate collection was at least once a week and on a more regular basis. It was observed that since 2017, the frequency of collection has been on a decline because of the insufficient number of workers and vehicles available for waste collection. This situation has also been compounded by limited funds as well as the prevailing socioeconomic problems facing the South and North West Regions of Cameroon (Awasom 2020).

Sakijege (2019) revealed that waste collection stations were not served with waste containment facilities in Keko Machungwa, Tanzania; therefore the waste generators were carrying wastes in plastic bags and few in dustbins which were transferred to the collection points. In Limbe, because of the limitations in waste collection and the fact that the waste management system of the municipality does not provide waste storage containers to households, the type of storage device is strongly dependent on individual households. The geographical relief of Limbe results in the emergence of short duration, fast running bodies of water from peripheral areas towards the mainland and onwards into the Atlantic Ocean. Thus, wantonly disposed waste and other debris materials constitute a major load for such running water bodies, consequently blocking water channels. This triggers overland flows and contributes to floods. This situation is quite similar to what Tvedten and Candiracci (2018) observed in the city of Maputo. Despite the fact that the Maputo Municipal Council (CMM) had provided basic services, including waste collection in both the formal and informal parts of the city, inefficiencies and resource constraints limited the ability of the Council to provide quality MSWM services to its citizens. This has been exacerbated by the fact that the city of Maputo has a very limited municipal tax base, with property registers either outdated or non-existent, and generally no culture of tax payment. However, the challenges in MSWM in Maputo is different from that in Limbe, as it rests on inadequate understanding and communication between two very different waste management perspectives: a Western and/or neoliberal and formal system of collection on the one hand and the experiences, needs and perspectives of people in the informal settlements on the other hand (Tvedten & Candiracci 2018). Municipal solid waste management in developing countries has been and will continue to be problematic because many governments are not able to meet all the needs of the rapidly growing population (Sakijege 2019).

Within the past 4 years, the sociopolitical crisis has given rise to a new phenomenon in waste management in the city of Limbe – the proliferation of huge unauthorised piles of waste on the ground surface along collection routes. This outcome is a consequence of several factors including the absence or limitation in number of collection vehicles, unavailability of workers because of safety issues and distance from households to any apparent collection points.

Contrary to the situation in the city of Limbe (Cameroon) where waste disposal is solely the responsibility of the municipal council and a contracted agent – a non-governmental organisation (NGO), the situation is different in other cities in Africa like Dar es Salaam in Tanzania (Sakijege 2019). The Tanzanian model employs a public-private sector partnership in MSWM. In the case of Limbe, primary collection of households is done by generators, that is households and institutions while collection, transportation and disposal of MSW is done by the NGO contracted by the council. In the case of Dar es Salaam, the private entities have been used to complement the inadequate capacity of the public sector. In Keko Machungwa (a locality that hosts 4180 houses) in Dar es Salaam, Tanzania, waste collection and transportation is being done by an established women participatory group instituted in 2018. This group is made up of a total of 8 permanent and 40 temporal members who work for 10 h a month with a threefold responsibility: (1) to collect waste collection fees; (2) to encourage and insist people to transfer their wastes to the waste collection points and (3) to clean waste residual at the waste collection points after being transferred to the trucks (Sakijege 2019). Thus, the expansion of the Limbe model to incorporate aspects of the Dar es Salaam model may be very valuable in serving peripheral areas not covered by the lone contracted service of the council. It is important that various stakeholders be brought on board for full and active participation. However, the level of stakeholder participation is dependent on a number of factors, one of them being the level of awareness and information on waste management by the local population.

The practice in Limbe whereby households are responsible for transferring their generated waste to collection points is similar to that in Keko Machungwa locality in Dar es Salaam (Sakijege 2019). In Keko Machungwa, however, those who accept to pay the collection fee had no obligations of carrying their waste to the collection points. In Keko Machungwa, the collection points are selected based on the accessibility to the means of transport. As it is in Limbe, a larger part of Keko Machungwa is not planned and as such cannot easily be accessed. This resulted to absence of waste collection points. However, contrary to Limbe where residents in localities without collection points dispose of their wastes in streams or burn them, residents of Keko Machungwa without waste collection points carry their waste to the collection points, which in turn simplifies waste collection. Thus, there is a comparatively lower willingness to participate in proper waste management by residents in peripheral and poorly planned areas in Limbe than in Keko Machungwa.

The level of stakeholder participation in MSWM is dependent on a number of factors. An important factor in this case is the level of awareness and information on waste management by the local population. In this study, it is observed that a greater majority of respondents are not linked to any formal information medium on waste management. An implication of this on the waste management system may be that you find individuals who engage in unsustainable practices simply because they are acting within the limits of their knowledge and convictions. A similar study carried out in Dar es Salaam showed a similar trend to ours that there is little awareness among the residents whereby a majority of respondents considered waste collection as a useless exercise, which implied they were not supposed to pay for it (Sakijege 2019). It should be noted that, as long as the level of awareness is low, residents do not find their individual preferences to management as problematic. For waste management to be very effective, the community involvement is crucial. This involvement implies that they should have some basic knowledge about the management system in operation and hence fully participate (Callan & Thomas 2001). Such a low value of knowledge on waste management by the public implies that given the declining services especially during this politically difficult moment, the public may be further engaging in wanton disposal practices.

For waste collection and transportation to be effective and efficient, the collection and transportation services have to be physically intact. This implies that from the level of generation to storage, collection and transportation, treatment and disposal, the waste must be sealed to reduce this interaction. Thus, the physical state of collection bins is critical in determining this interaction. Public waste collection bins are expected to remain closed at all times and to have wheels to ease rolling towards collection trucks in order to empty contents. In the observed cases, none of the bins was covered with only 28% having lids. In our study, we found out that a far greater number of collection facilities are not in good physical shape and transportation infrastructure is in shortage. This implies that despite the fact that councils are operating under tight budgets, time losses in operations as a consequence of poor physical state of bins further constrain the waste management system of the municipality. In Dar es Salaam, similar to Limbe, the city used municipal trucks to transport collected solid waste to disposal sites. However, here, truck operators were responsible for delivering wastes to the truck ready for transportation to designated disposal sites from waste collection stations once a week (Sakijege 2019). However, just like the situation in Limbe, delays have been observed linked to the size of the car and volume of the solid wastes generated, which makes it impossible to transport wastes in a single trip and adds to unnecessary costs. Delays in transporting wastes to the landfill as observed in Limbe and similarly in Keko Machungwa potentially lead to environmental hazards in the surrounding environment and increase flood risk.

Annually, the city of Limbe witnesses some degree of floods. Both natural and anthropogenic factors account for these floods, and the associated impacts have been inundation of buildings, disruption of services, disease outbreaks and human deaths (Lambi et al. 2002; Ndille & Belle 2014; Wantim et al. 2022). By 2018, rapid increase in population and urban sprawl had started constraining the effectiveness of the waste collection system. More so, the state of collection vehicles has not been the best in terms of age and functionality. Studies carried out by Salami et al. (2017) revealed that lack of basic knowledge and understanding of flood risk by the people living in flood-prone areas, poor governance and social and environmental injustice were the underlying causes of flood risk. For instance, a city with a very low quality of basic infrastructure services, unplanned growth and rapid urbanisation coupled with effects of climate change can turn a heavy rainfall into a catastrophic flood. Most cities and urban centres in Africa are regarded as flood disaster risk hotspots because of rapid urbanisation which has led to the encroachment of the population on flood-prone zones as was revealed in the study by Ramiaramanana and Teller (2021) in the city of Antananarivo in Madagascar.

The recurrent floods experienced by the city of Limbe since 2001 can be significantly linked to the poor MSWM practices. This finding is supported by studies carried out by Mokuolu et al. (2022) which highlighted the fact that municipal and domestic wastes are most likely the most dominant category of urban waste which in most instances are dumped into surrounding drains and streams because of lack of waste collection services. Studies by Ziraba, Haregu and Mberu (2016) have shown that open dumpsite inside drains or rivers, as is the case in some residential areas in Limbe city, increases the pressure on the already overburdened drainage system, causing urban floods to become more frequent and severe. Additionally, the fact that waste management in Limbe is the sole responsibility of the municipal council, where the local administrators are already overburdened with other developmental issues for the municipality, makes the task of proper waste management to become difficult because of competing priorities for the limited financial resources/budget to be managed by the council. With this state of affairs, there is bound to be more recurrent floods in the city of Limbe if other stakeholders are not engaged in MSWM. However, even with the engagement of third parties such as NGOs, the effects are usually short-termed as revealed by Mokuolu et al. (2022). Awareness raising and sensitisation of the public remains the best solution to proper waste management and curbing flood risk in urban areas in sub-Saharan Africa. This aspect was recently highlighted in the January 2023 floods that hit the city of Kinshasa in the Democratic Republic of Congo (Ntumba 2023). This flood was attributed to poor waste management practices by the residents of Kinshasa who dispose of their waste in any location and even go ahead to ignore the garbage bins provided by the municipality resulting to clogged gutters and rivers which ultimately resulted to floods. Awareness raising and sensitisation will help the population realise that their own waste is their greatest adversary. The implications of this on the waste management system may be that you find individuals who engage in unsustainable practices simply because they are acting within the limits of their knowledge and convictions.

## Conclusions

In the city of Limbe, primary waste collection is done by the public while secondary waste collection is done by the LCC. The results of this study show that the strategies – short-term, mid-term and long-term strategies – put in place by the LCC to manage floods and waste have significant benefits considering environmental, socioeconomic and technical aspects. Thus, the council should continue actions within the three strategies. However, despite the successes, there are aspects that still have negative impacts on flood management. A major determinant with negative outcome is cost. This study further revealed that a greater majority of the population in the investigated neighbourhoods are not linked to any formal information medium on waste management, thus the gross abuse in this sector. Both natural and anthropogenic factors account for floods in Limbe, exacerbated by population growth and an ineffective waste management system.

### Recommendations

The LCC should introduce recycling programmes that may offset part of waste disposal cost. The key areas to be given greater attention include improvement and upgrading of waste collection services, enhancement of public knowledge and participation in waste management.
